# Antibiotic fosmidomycin protects bacteria from cell wall perturbations by antagonizing oxidative damage-mediated cell lysis

**DOI:** 10.3389/fmicb.2025.1560235

**Published:** 2025-04-16

**Authors:** Yoshikazu Kawai, Jeff Errington

**Affiliations:** Faculty of Medicine and Health, University of Sydney, Sydney, NSW, Australia

**Keywords:** *Bacillus subtilis*, cell wall, isoprenoid, oxidative damage, fosfomycin, fosmidomycin

## Abstract

Cell wall peptidoglycan is a defining component of bacterial cells, and its biosynthesis is a major target for medically important antibiotics. Recent studies have revealed that antibiotics can kill cells not only by their direct effects on wall synthesis, but also by downstream perturbations of metabolic homeostasis, leading to oxidative damage-mediated lysis. In this paper, we have investigated the killing effects of various effectors of cell wall inhibition, including an antibiotic inhibitor of isoprenoid synthesis, fosmidomycin, in *Bacillus subtilis*. We show that oxidative damage largely contributes to the toxic effect (rapid cell lysis) induced by inhibition of peptidoglycan synthesis, but not by inhibition of the isoprenoid synthetic pathway. Remarkably, intermediate concentrations of fosmidomycin, confer resistance to lysis when peptidoglycan synthesis is perturbed. We show that this is because fosmidomycin not only blocks peptidoglycan synthesis, but also impairs the synthesis of menaquinone, which, protects cells from respiratory chain-associated oxidative damage and lysis. Our results provide new insights into the critical involvement of metabolic pathways, such as isoprenoid biosynthesis, on the antibiotic efficacy and evasion by bacteria. This work advances our understanding of bacterial physiology as well as antibiotic activity and resistance.

## Introduction

The cell wall is essential for bacterial growth and viability. The wall constitutes a major proportion of the mass of the cell (over 60%) in Gram-positive bacteria (Silhavy et al., 2010), maintains the characteristic shape of the cell, and protects cells from fluctuations in their internal osmotic pressure. The major component of the wall is peptidoglycan (PG), which comprises glycan strands cross-linked by short peptides, forming a huge contiguous meshwork that covers the whole surface of the cell (Höltje, 1998). The PG precursor molecule, called lipid II, is made on the cytoplasmic face of the cell membrane through a well conserved biochemical pathway, then flipped to the outside of the cytoplasmic membrane. New lipid II is inserted into the existing PG wall by the action of glycosyltransferase (GTase) and transpeptidase (TPase) enzymes, enabling expansion of the growing cell (Egan et al., 2020). PG synthesis remains an outstanding target for existing and future antibiotic therapeutics.

*β*-Lactams are one of the oldest and still most widely used clinical antibiotics, which prevent the insertion of new lipid II by binding covalently to the TPase domains of penicillin binding proteins (PBPs) (Lovering et al., 2012). Fosfomycin (FOS) inhibits the enzyme, MurA, which catalyses the first step in the lipid II synthetic pathway (Silver, 2017). It has been generally assumed that the gross morphological changes that often accompany treatment with cell wall-active antibiotics (and mutations perturbing cell wall synthesis) are the main cause of subsequent lysis and cell death, through loss of cell wall integrity (Schwarz et al., 1969; Klainer and Perkins, 1970). However, recent studies on a range of bacteria have revealed that perturbations of cell wall metabolism can affect downstream metabolic pathways, leading to oxidative damage and sometimes death (Kohanski et al., 2007; Yeom et al., 2010; Cho et al., 2014; Belenky et al., 2015; Dwyer et al., 2015; Kawai et al., 2015; Kawai et al., 2019; Leger et al., 2019; Stokes et al., 2019; Shin et al., 2021; Kawai et al., 2023). Hence, antibiotic efficacy can be influenced by the growth conditions or metabolic state of the bacterium (Stokes et al., 2019).

In the rod-shaped Gram-positive bacterium *Bacillus subtilis*, genetic dissection of cell wall damage and cell lysis has recently revealed the key metabolic steps that stimulate the generation of reactive oxygen species (ROS) through central carbon oxidation pathways (i.e., glycolysis, TCA cycle and the respiratory chain) (Kawai et al., 2023). It is generally thought that the increased generation of ROS damages various important macromolecules, especially lipid peroxidation, catalyzed by redox-active iron (Imlay, 2003; Lushchak, 2014). Membrane damage leads to cell lysis and death (Ayala et al., 2014), as evident from a phase pale or “ghost” microscopic appearance due to leakage of cell components (Reuter et al., 2014; Kawai et al., 2018; Pospisil et al., 2020; Kawai et al., 2023). Reducing the generation of ROS by compensating for the harmful metabolic shifts or chelating iron can specifically prevent the lytic effect, while morphological damage (e.g., bulging or twisting), often associated with lysis, is unaffected (Kawai et al., 2023). It thus appears that cells suffer from two phenotypic abnormalities upon perturbation of cell wall synthesis – shape changes and a phase pale appearance (lysis) – and that these effects arise by distinct mechanisms.

**Figure 1:**
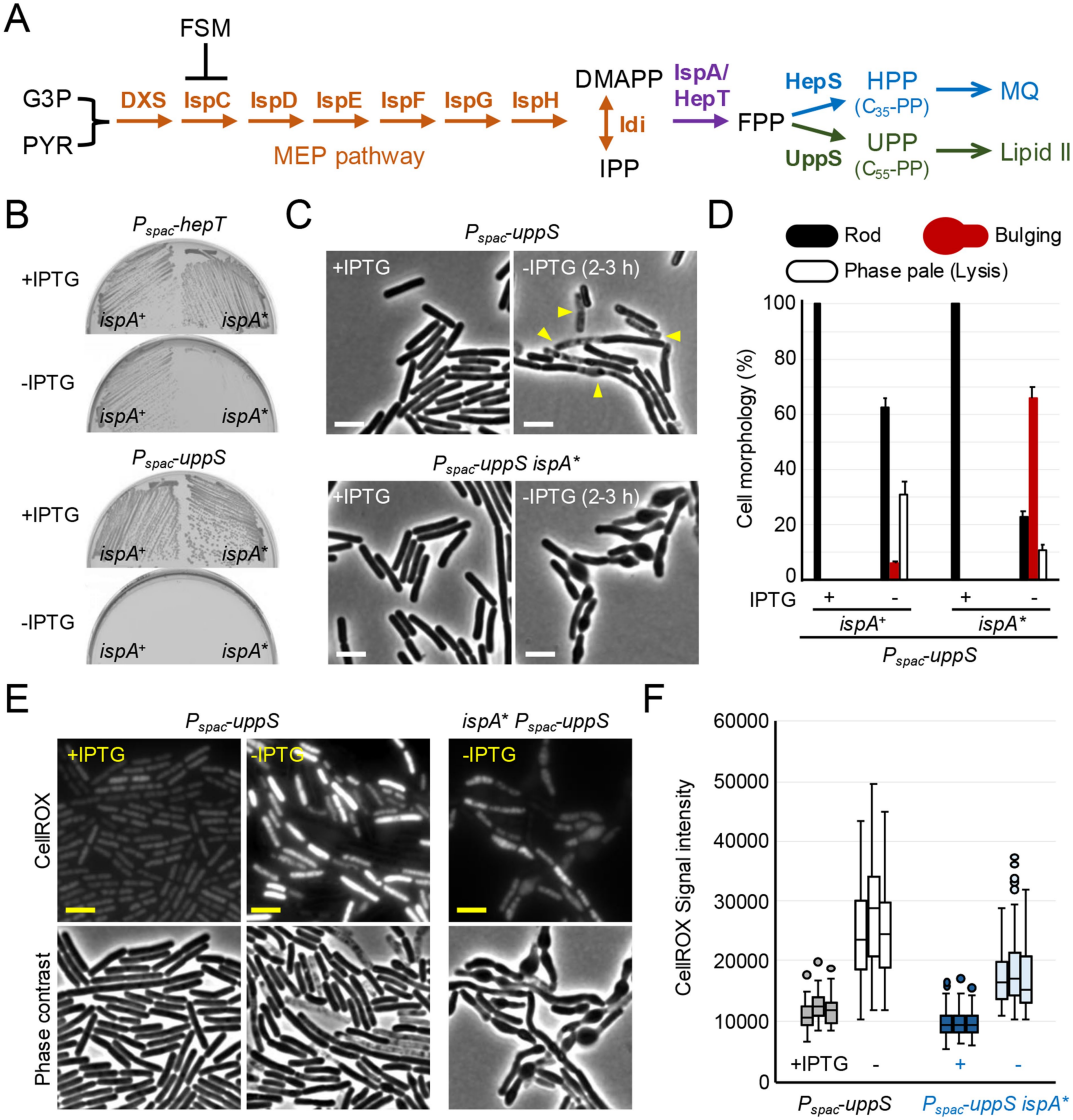
ROS-mediated lysis through inhibition of UPP synthesis. **(A)** Schematic representation of isoprenoid biosynthesis in *B. subtilis*. The methylerythritol phosphate (MEP) pathway involves seven reaction steps to produce dimethylallyl pyrophosphate (DMAPP) and isopentenyl pyrophosphate (IPP) from the glycolytic intermediates, glyceraldehyde 3-phosphate (G3P) and pyruvate (PYR). IspA and HepT catalyze the formation of farnesyl pyrophosphate (FPP) precursor, which is used to form two lipid molecules: heptaprenyl pyrophosphate (HPP) for menaquinone (MQ) and undecaprenyl pyrophosphate (UPP), the lipid carrier moiety of lipid II. Fosmidomycin (FSM) inhibits IspC in the MEP pathway. **(B)** Growth of *B. subtilis* strains; YK1451 (*P_spac_*-*hepT*), YK1453 (*P_spac_*-hepT ispA*), YK1429 (*P_spac_*-*uppS*) and YK2821 (*P_spac_*-uppS ispA*) on NA plates with or without 0.5 mM IPTG. The ispA* mutation represents an *ispA* allele with reduced expression levels, ispA* (Mercier et al., 2013; Kawai et al., 2015). **(C)** Phase contrast micrographs of YK1429 (*P_spac_*-*uppS*) and YK2821 (*P_spac_*-uppS ispA*). The exponentially growing cells in liquid NB with 0.2 mM IPTG (+IPTG) were diluted into fresh NB (-IPTG) and incubated for 3 h. The yellow arrowheads indicate a phase pale appearance (lysis). Scale bars represent 5 μm. **(D)** The cell morphologies shown in panel C were classified into three types (Rod, Bulging or Phase pale). 150–200 cells were analyzed for each condition. Error bars represent the standard deviation of three independent experiments. **(E)** Phase contrast and the corresponding fluorescence images (CellROX-green) of YK1429 (*P_spac_*-*uppS*) and YK2821 (*P_spac_*-uppS ispA*). The exponentially growing cells in liquid NB with 0.2 mM IPTG (+IPTG) were diluted into fresh NB (-IPTG) and incubated for 3 h. Culture samples were treated with CellROX-green for 30 min and washed before imaging. Scale bars represent 5 μm. **(F)** The signal intensity of green fluorescence in the cells shown in panel **(E)** was plotted as boxplots (*n* ≈ 90). Boxplots represent the upper and lower quartile values (boxes), the median (horizontal lines in the boxes) and the most extreme data points within 1.5 times the interquartile ranges (whiskers). Dots represent outliers. The experimental data in this figure are representative of at least three independent experiments.

Isoprenoids are ubiquitous in living organisms and possess diverse structures and essential biological functions (Gershenzon and Dudareva, 2007), including lipid II synthesis and cellular respiration. The methylerythritol phosphate (MEP) pathway is responsible for the synthesis of isoprenoids in many bacteria, including *B. subtilis* and *Escherichia coli*, but not in humans (Lange et al., 2000; Kuzuyama, 2002), and is therefore a potential target for antimicrobial drug development. The MEP pathway (Figure 1A) generates isoprenoid lipid precursors, dimethylallyl pyrophosphate (DMAPP) and isopentenyl pyrophosphate (IPP), which are used to synthesize farnesyl pyrophosphate (FPP). FPP is then used for the synthesis of bactoprenol (undecaprenyl pyrophosphate: UPP), the lipid carrier moiety of lipid II, and HPP (heptaprenyl pyrophosphate) for menaquinone, a central player in the respiratory chain. Our previous results have shown that downregulation of menaquinone synthesis counteracts the oxidative damage-mediated lytic effect upon cell wall perturbations by reducing ROS production from the respiratory chain (Kawai et al., 2015; Kawai et al., 2023).

Here, we show that inhibition of UPP synthesis exhibits ROS-mediated lytic effects in *B. subtilis*. These effects can be suppressed by downregulating the synthesis of the UPP precursor, FPP. Furthermore, we show that sub-inhibitory concentration of another antibiotic, fosmidomycin (FSM), an inhibitor of the MEP pathway, can antagonize the lytic effect induced by FOS. The antagonistic effect appears to depend on a reduced supply of the precursors for menaquinone synthesis from the MEP pathway. Our results provide new insights into how specific physiological changes influence antibiotic efficacy.

## Materials and methods

### Strains and growth conditions

The bacterial strains used in this study are listed in Table 1. DNA manipulations and transformations were carried out using standard methods. Nutrient agar (NA) and broth (NB) (Oxoid) were used for bacterial growth of walled cells at 37°C. *B. subtilis* L-forms (strain YK1846) were cultured in isotonic NB medium (NB/MSM), consisting of 2x magnesium-sucrose-maleic acid (MSM) pH 7.0 (40 mM magnesium chloride, 1 M sucrose, and 40 mM maleic acid) mixed 1:1 with 2x NB, at 30°C without shaking (Leaver et al., 2009; Mercier et al., 2013). For selections of *B. subtilis* mutants, antibiotics were added to the media at the following concentrations: 1 μg mL^−1^ erythromycin, 5 μg mL^−1^ chloramphenicol, 60 μg mL^−1^ spectinomycin or 5 μg mL^−1^ kanamycin (15 μg mL^−1^ kanamycin in the presence of 10 mM Mg^2+^). IPTG and or 10 mM MgSO_4_ (for Mg^2+^) were supplemented, as appropriate. Fosfomycin and fosmidomycin (Cayman Chemical) were used as indicated. Mirubactin C used in this study were previously purified or synthesized (Kepplinger et al., 2022).

**Table 1:** *Bacillus subtilis* strains.

Strains	Genotypes	References
168CA (wild-type)	*trpC2*	Lab. stock
YK1343	*trpC2 rodA*::*neo*	Kawai et al. (2011)
YK1395	trpC2 ispA^*^ (*xseB*::*Tn*-*kan*)	Mercier et al. (2013)
YK1424	*trpC2* Ω*ispA*::*erm*-*P_spac_*-*ispA*	Kawai et al. (2015)
YK1425	*trpC2* Ω*dxs*::*erm*-*P_spac_*-*dxs*	This work
YK1426	*trpC2* Ω*ispD*::*erm*-*P_spac_*-*ispD*-*ispF*	This work
YK1429	*trpC2* Ω*uppS*::*erm*-*P_spac_*-*uppS*	This work
YK1451	*trpC2* Ω*hepT*::*erm*-*P_spac_*-*hepT*	This work
YK1453	*trpC2* Ω*hepT*::*erm*-*P_spac_*-hepT ispA*	This work
YK1540	*trpC2* Ω*murG*::*erm*-*P_spac_*-*murG*-*murB*	Kawai et al. (2023)
YK1846^†^	*trpC2 ∆18*::*tet*	Kawai et al. (2014)
YK2638	*trpC2 ∆mbl*::*cat*	Schirner and Errington (2009)
YK2684	*trpC2 ∆mbl*::*cat* Ω*dxs*::*erm*-*P_spac_*-*dxs*	This work
YK2685	*trpC2 ∆mbl*::*cat* Ω*ispD*::*erm*-*P_spac_*-*ispD*-*ispF*	This work
YK2820	*trpC2* Ω*bshB1*::*erm*-*P_spac_*-*bshB1-bshA*	Kawai et al. (2015)
YK2821	*trpC2* Ω*uppS*::*erm*-*P_spac_*-uppS ispA*	This work

^†^The L-form strain contains an 18 kbp deletion, (missing the murC gene which encodes an essential enzyme in the lipid II synthesis, together with 17 other coding regions).

### Construction of IPTG-inducible *Bacillus subtilis* mutants

To construct the IPTG-inducible *dxs*, *ispD-ispF*, *uppS* and *hepT*, the first 200–300 bp of the genes or operons containing the Shine-Dalgarno sequence were amplified by PCR from the genomic DNA of the *B. subtilis* 168CA strain and then inserted into plasmid pMutin4 (Vagner et al., 1998). The resulting plasmids were introduced into the *B. subtilis* 168CA via Cambell recombination to generate strains YK1425, YK1426, YK1429 and YK1451 (Table 1). The sequences of the primers used for strain construction are listed in Table 2.

**Table 2:** Primers.

dxs-F	GAAGAATTC TGAAAGTGAGTTGATCCG
dxs-R	GGAGGATCC GTTTCCCAAACATCGTGC
ispD-F	GAAGAATTC CTGTAAAGGGAGAAGAAAC
ispD-R	GGAGGATCC GTTTGAAACGGGTAATCGG
uppS-F	GAAGAATTC TTGGGTGACGGAGGAATC
uppS-R	GGAGGATCC CATTAAAAAATCGACCTCC
hepT-F	GAAGAATTC CAGGCCTGTTTGAAGAGG
hepT-R	GGAGGATCC AATCGCCAAACATGCCAG

### Disk diffusion assay

*Bacillus subtilis* cells were grown to OD_600_ of 0.6 in NB with appropriate requirements. Cells (100 μL of the cultures) were mixed with 50 mL of molten NA supplemented with appropriate requirements and plated. Whatman Antibiotics Assay Discs (6 mm diameter) were used for the assays.

### Detection of ROS

*Bacillus subtilis* strains were cultured in NB with appropriate requirements at 37°C. To detect ROS (superoxide and hydroxyl radical), 0.5 mL of the cultures in 2 mL microtubes were incubated with 2.5 μM CellROX Green (Thermo Fisher Scientific) for 30 min at 37°C. The cells were harvested by centrifugation and washed three times with fresh NB before being used for microscopic analysis. CellROX Green is a proprietary oxidation-sensitive dye whose fluorescence quantum yield at 500–550 nm after excitation at 488 nm increases dramatically upon oxidation in the presence of dsDNA (Choi et al., 2015).

### Microscopy and image analysis

For live cell snapshot imaging, bacterial cells were mounted on microscope slides covered with a thin film of 1.2% agarose in water, or in NB/MSM for L-forms. All microscopy experiments were performed on a Nikon Inverted Research Microscope ECLIPSE Ti2 equipped with a Nikon CFI Plan Apochromat DM Lambda 100x oil objective and a Teledyne Photometrics Prime BSI camera, using a Nikon NIS-Elements AR software. Images were analyzed and processed using FIJI (version 2.9.0/1.53 t^1^).

## Results

### ROS-mediated lysis through inhibition of UPP synthesis

Synthesis of the FPP precursor, used for UPP and HPP synthesis, is controlled by two genes, *hepT* and *ispA* (Figure 1A), which appear to be partially redundant, as *ispA* is non-essential (Mercier et al., 2013; Koo et al., 2017). Indeed, Figure 1B shows that the combination of a previously described *ispA* allele with reduced expression levels, ispA* (Mercier et al., 2013), and depletion of *hepT* using an IPTG-inducible *P_spac_* promoter, resulted in growth arrest on nutrient agar (NA) plates in the absence of IPTG.

**Figure 2:**
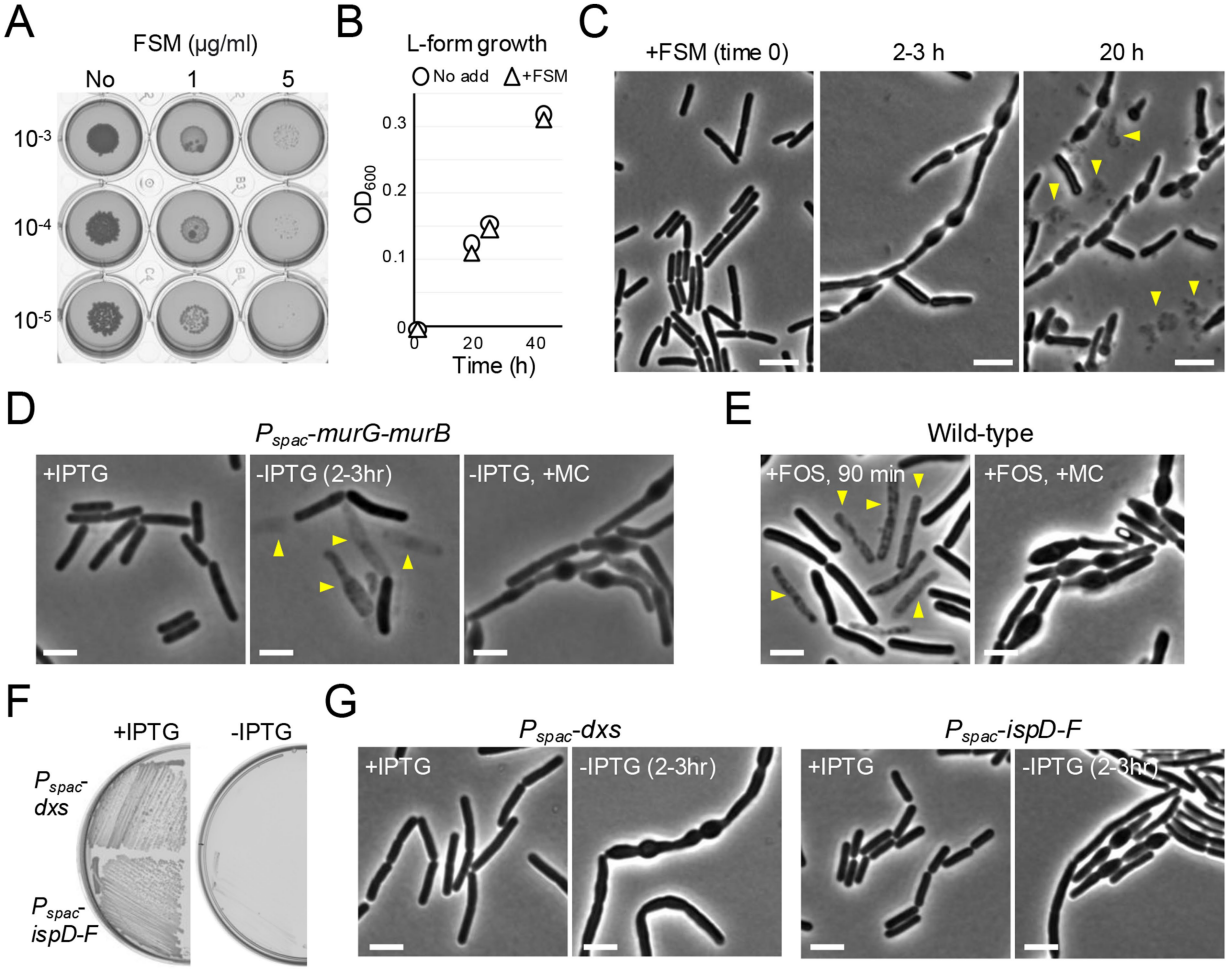
Inhibition of the MEP pathway generates morphological change without immediate lysis. **(A)** Exponentially growing wild-type (168CA) cells in NB were diluted (tenfold series) and 6 μL spots were placed on NA plates with (1 or 5 μg mL^−1^) or without FSM. **(B)** Growth of *B. subtilis* L-forms (YK1846) in NB with osmo-protective solution MSM. L-forms were diluted in fresh NB/MSM with or without 5 μg mL^−1^ FSM and OD_600_ was measured. **(C)** Phase contrast micrographs of wild-type (168CA). The exponentially growing cells in NB were treated with 5 μg mL^−1^ FSM. Scale bars represent 5 μm. **(D)** Phase contrast micrographs of YK1540 (*P_spac_*-*murG-murB*). The exponentially growing cells in liquid NB with 0.2 mM IPTG (+IPTG) were diluted into fresh NB with or without 10 μg mL^−1^ Mirubactin C (MC) and incubated for 3 h. The yellow arrowheads indicate a phase pale appearance (lysis). Scale bars represent 5 μm. **(E)** Phase contrast micrographs of wild-type *B. subtilis* (168CA). The exponentially growing cells in NB were treated with or without 10 μg mL^−1^ FOS and 10 μg mL^−1^ MC and incubated for 90 min. The yellow arrowheads indicate a phase pale appearance (lysis). Scale bars represent 5 μm. **(F)** Growth of *B. subtilis* strains; YK1425 (*P_spac_*-*dxs*) and YK1426 (*P_spac_*-*ispD-ispF*) on NA plates with or without 0.5 mM IPTG. **(G)** Phase contrast micrographs of YK1425 (*P_spac_*-*dxs*) and YK1426 (*P_spac_*-*ispD-ispF*). The exponentially growing cells in liquid NB with 0.2 mM IPTG (+IPTG) were diluted into fresh NB (-IPTG) and incubated for 3 h. Scale bars represent 5 μm. The experimental data in this figure are representative of at least three independent experiments.

The UppS enzyme generates UPP for lipid II synthesis, and the HepS enzyme generates HPP for menaquinone synthesis (Figure 1A). To specifically test for a block in UPP synthesis, the *uppS* gene was placed under the control of *P_spac_*. Figure 1B shows that the strain carrying *P_spac_-uppS* did not grow on NA plates in the absence of IPTG, confirming that *uppS* is an essential gene (Inaoka and Ochi, 2012). In liquid nutrient broth (NB) with IPTG, the cells had a typical rod-shaped morphology (Figure 1C) and were indistinguishable from the parental wild-type cells (see below). However, in the absence of IPTG, lysis (phase paling) occurred but without evident shape changes (although occasional minor bulging) (Figures 1C,D), consistent with ROS-mediated lysis when lipid II synthesis was blocked by repressing expression of the *murG*-*murB* operon (Kawai et al., 2023) (see also Figure 2D).

We have previously shown that partial inhibition of HPP synthesis, either by downregulation of the *hepS* or *ispA* genes (Figure 1A), can counteract ROS-mediated lysis upon cell wall perturbation, by reducing ROS production from the respiratory chain (Kawai et al., 2015; Kawai et al., 2023). We then anticipated that the ispA* mutation might protect UppS-depleted cells from lysis. As shown in Figures 1C,D, when *uppS* was depleted in the presence of the ispA* mutation, immediate cell lysis was blocked and cells instead developed a bulging phenotype. CellROX-Green (a fluorescent dye) for detection of superoxide and hydroxyl radical (Choi et al., 2015; McBee et al., 2017; Gibson et al., 2021) staining of the cells directly showed that ROS levels were greatly increased following UppS depletion, while this was significantly reduced by the ispA* mutation (Figures 1E,F).

These results are consistent with the notion that immediate cell lysis upon lipid II inhibition is largely mediated by ROS and support our previous findings that morphological perturbations and cell lysis can be distinct, separable phenomena (Kawai et al., 2023). It also suggests that the lytic effect is dependent on a supply of FPP precursors for the HPP pathway.

### Inhibition of the MEP pathway generates morphological change without immediate lysis

FSM is a broad-spectrum antibiotic, the first representative of a new class of antimalarial drugs that inhibits deoxyxylulose 5-phosphate (DXP) reductoisomerase, IspC, in the MEP pathway (Figure 1A; Kuzuyama et al., 1998; Zeidler et al., 1998; Koppisch et al., 2002; Zhang et al., 2011; Yeh and DeRisi, 2011). Growth of *B. subtilis* on NA plates was severely impaired in the presence of 1 μg mL^−1^ FSM and virtually eliminated at 5 μg mL^−1^ (Figure 2A). In contrast, FSM (at 5 μg mL^−1^) did not affect growth of cell wall-free L-forms in liquid culture (Figure 2B); a state in which cells can proliferate and survive without PG synthesis (Leaver et al., 2009; Errington et al., 2016), suggesting that FSM primarily acts by targeting PG synthesis. Note that the L-forms (strain YK1846) used here contain an 18 kbp deletion, (missing the *murC* gene which encodes an essential enzyme in the lipid II synthesis, together with 17 other coding regions) (Mercier et al., 2013; Kawai et al., 2014). We then used phase contrast microscopy to examine the effects of FSM treatment on cell morphology in walled cells. Within 3 h of treatment with 5 μg mL^−1^ FSM, morphological damage with abnormal cell bulging was observed, but the cells remained phase dark (Figure 2C, 2–3 h), although with longer incubation many cells lysed (>20 h).

As controls, inhibition of lipid II synthesis by repressing expression of the *murG*-*murB* operon, or by treatment with FOS (which is a broad-spectrum bactericidal antibiotic that inhibits the first committed step of the lipid II pathway) (Silver, 2017), induced a rapid lysis (within 2 h) without significant morphological change (Figures 2D,E), just as seen for UppS depletion (Figure 1C). However, in the presence of our previously described iron chelator mirubactin C (MC) (Kepplinger et al., 2022), which can reduce the production and damaging effects of ROS (Kawai et al., 2023), the lytic effect by *murG*-*murB* repression and FOS treatment was alleviated, while many cells now began to bulge (Figures 2D,E). These results confirm that rapid cell lysis upon lipid II inhibition is associated with oxidative damage as previously described (Kawai et al., 2023).

Thus, inhibition of the MEP pathway by FSM appears to act primarily by blocking lipid II synthesis in *B. subtilis*, but the immediate effects differ from those of inhibition of UPP (or lipid II) synthesis in that cell lysis does not occur, consistent with the supply of the isoprenoid precursors for menaquinone synthesis being important for ROS-mediated lytic effects.

To test whether the effects of FSM were due to a specific inhibition of the MEP pathway, we constructed *P_spac_*-fusions to the *dxs* gene and the *ispD*-*ispF* operon, which encode enzymes in the MEP pathway (Figure 1A), and tested their effects on cell growth and morphology. Repression of these genes by removal of IPTG abolished growth on NA plates, confirming expectation that these genes are essential (Figure 2F). In liquid NB with IPTG, cells with *P_spac_-*fused to the *dxs* gene or the *ispD*-*F* operon were indistinguishable from wild-type cells (Figure 2G). When the culture was diluted into fresh NB without IPTG, bulging appeared upon depletion of Dxs or IspD-IspF (Figure 2G), but no significant lysis occurred for at least 4 h.

### Sensitivity to FOS is antagonized by FSM

**Figure 3:**
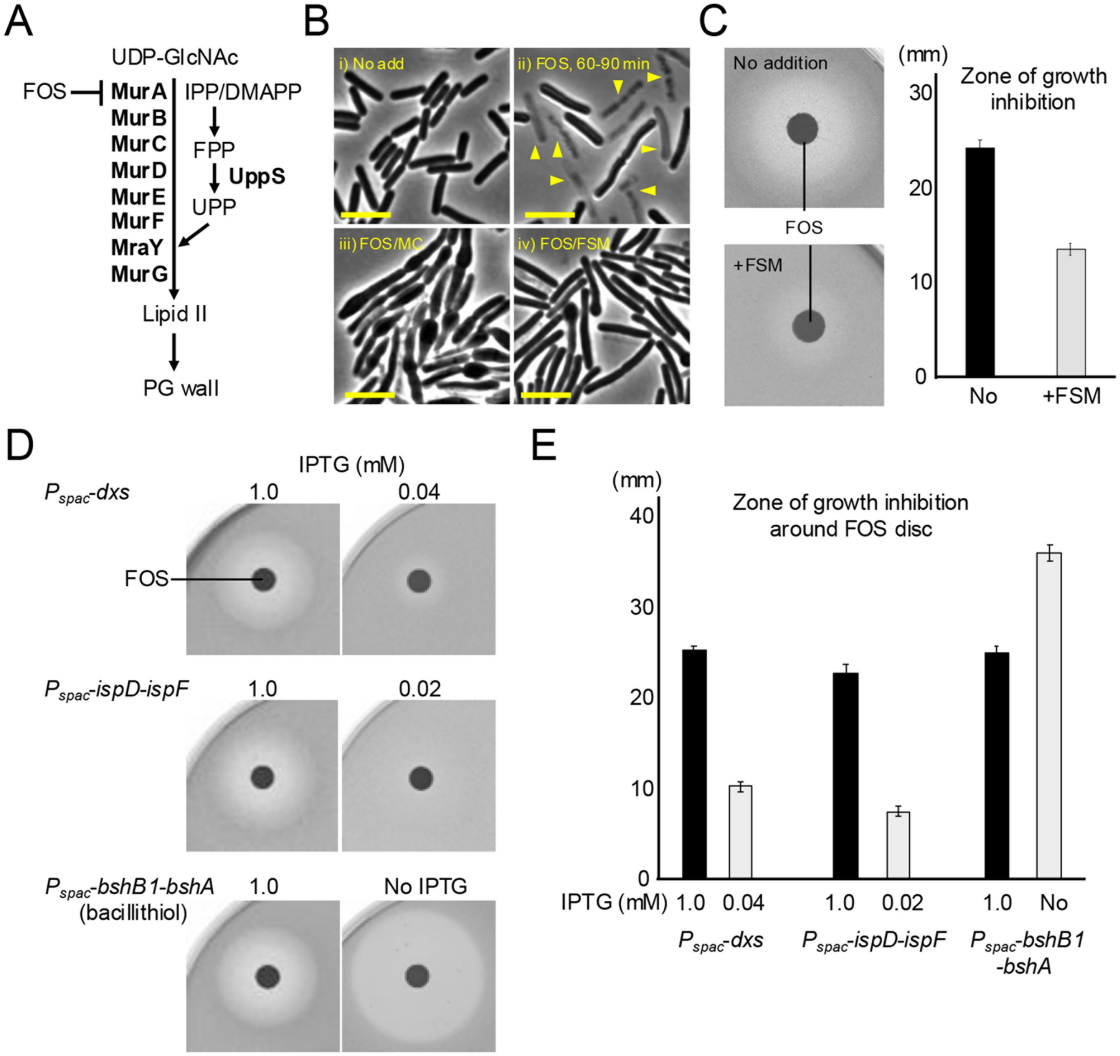
Inhibition of the MEP pathway confers resistance to FOS. **(A)** Schematic representation of lipid II synthesis in *B. subtilis*. Lipid II precursor is synthesized in the cytoplasm by a series of Mur enzymes and then attached to the lipid carrier UPP on the cytoplasmic face of the cell membrane by MraY, and the final step is catalyzed by MurG. Fosfomycin (FOS) inhibits MurA, which catalyses the first step in the lipid II pathway. **(B)** Phase contrast micrographs of *B. subtilis* wildtype (168CA). The exponentially growing cells in NB were treated with or without 10 μg mL^−1^ FOS, 10 μg mL^−1^ MC or 1 μg mL^−1^ FSM and incubated for 60–90 min. The yellow arrowheads indicate a phase pale appearance (lysis). Scale bars represent 5 μm. **(C)** Disk diffusion assay on NA plates using paper discs with 5 μL of 20 μg mL^−1^ FOS. Exponentially growing wild-type cells were plated on NA with or without 1 μg mL^−1^ FSM. Zone of growth inhibition was measured from four independent disc diffusion assay. The means and SD were shown. **(D)** Disk diffusion assay on NA plates using paper discs with 5 μL of 20 μg mL^−1^ FOS. Exponentially growing cells of strains YK1425 (*P_spac_*-*dxs*), YK1426 (*P_spac_*-*ispD-ispF*) and YK2820 (*P_spac_*-*bshB1*-*bshA*) in NB containing 0.2 mM IPTG were plated on NA with appropriate IPTG concentrations as indicated. The experimental data in this figure are representative of four independent experiments. **(E)** Zone of growth inhibition was measured from four independent disc diffusion assay. The means and SD were shown.

Given the above results, we were interested to test the effects of inhibition of the MEP pathway on the antibiotic action of FOS (an inhibitor of MurA in the lipid II pathway) (Figure 3A; Silver, 2017). Treatment with FOS in liquid medium induced a rapid phase paling effect (lysis) without significant morphological change (Figure 3Bii), as described above (Figure 2E). In the presence of iron chelator MC, the lytic effect was alleviated and many cells now began to bulge (Figure 3Biii). Similarly, when FOS treatment was combined with sub-inhibitory FSM (1 μg mL^−1^), the lytic effect was alleviated and cells began to bulge (Figure 3Biv).

Strikingly, disk diffusion assays confirmed that FSM treatment indeed clearly protected cells from the killing activity by FOS (much smaller zone of inhibition compared with no FSM treatment) (Figure 3C). Consistent with this, we found that partial repression of isoprenoid synthetic genes (*dxs* or the *ispD*-*ispF* operon) using *P_spac_*-fusions also conferred a degree of resistance to FOS. Upon reducing the expression of *dxs* or *ispD*-*ispF* with 0.04 or 0.02 mM IPTG, much smaller zones of growth inhibition were observed than with 1 mM IPTG (Figures 3D,E). As a control, FOS sensitivity was greatly increased when the synthesis of bacillithiol (Gaballa et al., 2010), an endogenous antioxidant that plays a role in FOS detoxification in several Firmicutes (Chandrangsu et al., 2018), was inhibited (Figures 3D,E).

These results infer that isoprenoid biosynthetic activity is critical for the ROS-mediated killing by FOS and an important potential route for resistance to lipid II inhibitors.

### FSM rescues mutants affected in the cell wall elongation Rod system

**Figure 4:**
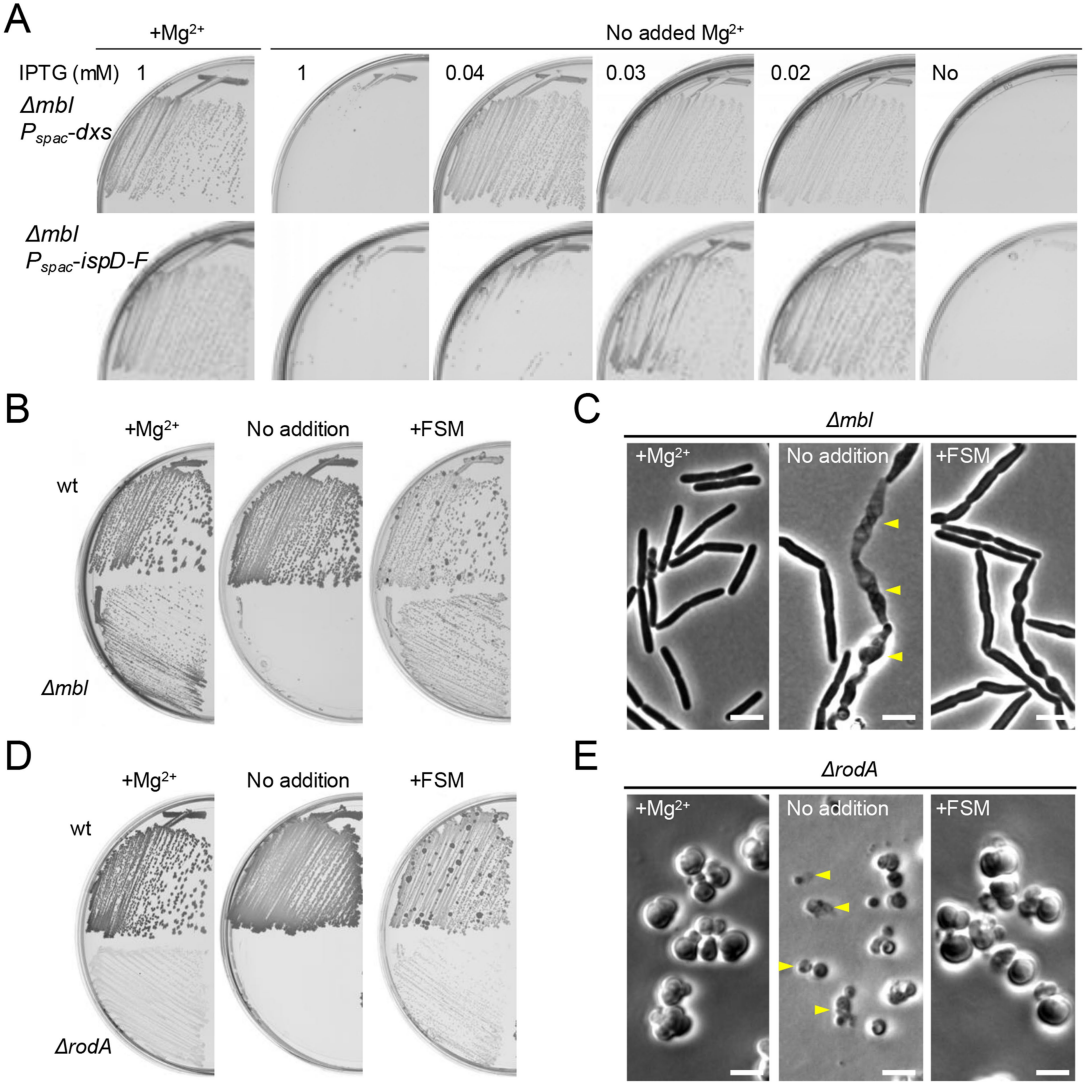
FSM rescues mutants affected in the cell wall elongation Rod system. **(A)** Growth of *B. subtilis* strains YK2684 (*Δmbl P_spac_*-*dxs*) and YK2685 (*Δmbl P_spac_*-*ispD-ispF*) on NA plates containing various amounts of IPTG (as indicated), with or without the addition of 10 mM Mg^2+^. **(B)** Growth of 168CA (wild-type) and YK2638 (*Δmbl*) on NA plates with or without the addition of 10 mM Mg^2+^ or 1 μg mL^−1^ FSM. **(C)** Phase contrast micrographs of YK2638 (*Δmbl*). The exponentially growing *Δmbl* cells in NB supplemented with 10 mM Mg^2+^ were diluted into fresh NB (without added Mg^2+^) and cultured with or without 1 μg mL^−1^ FSM for 120 min. The yellow arrowheads indicate a phase pale appearance (lysis). Scale bars represent 5 μm. **(D)** Growth of 168CA (wild-type) and YK1343 (*ΔrodA*) on NA plates (containing 100 mM NaCl for osmo-protection), with or without the addition of 10 mM Mg^2+^ or 1 μg mL^−1^ FSM. **(E)** Phase contrast micrographs of YK1343 (*ΔrodA*). The exponentially growing *ΔrodA* cells in NB (containing 100 mM NaCl) with added 10 mM Mg^2+^ were diluted into fresh NB (with 100 mM NaCl but without added Mg^2+^) and cultured with or without 1 μg/mL FSM for 120 min. The yellow arrowheads indicate a phase pale appearance (lysis). Scale bars represent 5 μm. The experimental data in this figure are representative of at least three independent experiments.

We have previously reported that mutations affecting PG synthesis in the cylindrical part of the cell, mediated by the Rod complex, can also cause ROS-mediated lysis (Kawai et al., 2023). The best characterized of these mutations affects a gene called *mbl*, which is an *mreB* paralogue that acts by spatially regulating elongation PG synthesis through the Rod system (Jones et al., 2001), and is normally essential for cell viability. However, lethality in an *mbl* mutant can be suppressed by preventing ROS production in several different ways: by reducing glycolysis or respiratory chain activity; or by chelating excess iron (Kawai et al., 2023). Although we do not yet understand the basis for protection by a high concentration of Mg^2+^, the effect provides a convenient way to enable growth of strains carrying the otherwise lethal mutation (Formstone and Errington, 2005; Kawai et al., 2009; Schirner and Errington, 2009). We introduced a *Δmbl* mutation into a strain carrying *P*_spac_-*dxs* or *P*_*spac*-_*ispD*-*ispF*. As expected, the *mbl* mutant cells grew well on NA plates supplemented with 1 mM IPTG (for *dxs* or *ispD*-*ispF* expression) and extra Mg^2+^ (for protection against ROS toxicity), but they failed to grow when Mg^2+^ was removed in the presence of 1 mM IPTG (Figure 4A). We then titrated the IPTG concentration to test for an effect of *dxs* and *ispD*-*ispF* expression levels on growth in the *mbl* mutant background. At lower IPTG concentrations (0.02–0.04 mM), the *mbl* mutant was able to grow even without additional Mg^2+^ (Figure 4A), consistent with expectation that reducing isoprenoid synthesis can overcome the ROS-mediated lethality of the *mbl* mutation. With no IPTG, growth was inhibited, now due to the complete loss of MEP pathway activity.

A sub-inhibitory concentration of FSM (1 μg mL^−1^) also rescued the growth of the *Δmbl* mutant in the absence of extra Mg^2+^ (Figure 4B). In a liquid NB culture with added Mg^2+^, *mbl* mutant cells showed a typical rod-shaped morphology (Figure 4C, left). When the culture was diluted into fresh NB without added Mg^2+^, the cells began to twist and bulge, and also began to lyse (Figure 4C, middle), as shown previously (Schirner and Errington, 2009). However, in the presence of FSM, the cells had similar morphological abnormalities, but they did not become phase pale (i.e., lysed) (Figure 4C, right), explaining the growth rescue effect on plates (Figure 4B).

The RodA GTase is a key synthetic enzyme in the Rod system, required for the cylindrical expansion of cell wall PG (Meeske et al., 2016; Emami et al., 2017; Sjodt et al., 2020). Just as for the *mbl* gene, *rodA* mutants can be rescued by chelating excess iron or adding high concentrations of Mg^2+^ (in the presence of osmolytes such as sucrose or NaCl) (Kawai et al., 2011; Kawai et al., 2023), although growth is slow and sustained only by the alternative aPBP system, which inserts PG in a disorganized manner, generating large pleomorphic, or spherical cells (Meeske et al., 2016; Emami et al., 2017). When the extra Mg^2+^ was removed, the *ΔrodA* mutant failed to grow on agar (Figure 4D), and in liquid culture *rodA* mutant cells were also spherical but small and frequently lytic (Figure 4E, middle). In contrast, in the presence of a sub-inhibitory concentration of FSM the cells continued to grow and divide in a manner very similar to that of the Mg^2+^-supplemented cells (Figures 4D,E). Thus, inhibition of the MEP pathway can prevent ROS-dependent lethality when elongation PG synthesis is blocked.

## Discussion

The role of downstream cellular metabolism and oxidative stress in the killing effects of, or resistance to, cell wall-active antibiotics has been investigated in a range of different bacteria (Kohanski et al., 2007; Yeom et al., 2010; Belenky et al., 2015; Kawai et al., 2019; Leger et al., 2019; Stokes et al., 2019; Shin et al., 2021; Kawai et al., 2023). In this report, we have investigated the effects of the antibiotics FOS and FSM, which act primarily by blocking lipid II synthesis, on oxidative damage-mediated cell death in *B. subtilis*. Our results showed that the killing activity of FOS was largely accompanied by a lytic effect. However, FSM treatment did not significantly induce this effect. Furthermore, FSM could protect cells from lysis by FOS. We dissected the steps linking the target of FSM in the MEP pathway to antagonize cell lysis by FOS (or inhibition of lipid II synthesis), and established that it works by restricting the availability of FPP and its precursors used for menaquinone synthesis. A growth rescue effect of FSM was also observed in strains affected in cell wall assembly, lacking the *rodA* or *mbl* genes, alleviating cell lysis and separating this effect from the severe morphological abnormalities arising from aberrant wall synthesis. These results support the idea that there are at least two distinct pathways potentially leading to cell death in the presence of cell wall-active antibiotics; ROS-dependent and ROS-independent (Kawai et al., 2023; Cho et al., 2014; Lobritz et al., 2022).

The ability of bacteria to survive antibiotic treatment is closely associated with chronic or recurrent infections. Resistance to FOS treatment has been mainly attributed to modification of the target, MurA, reducing its cellular accumulation or the acquisition of antimicrobial resistance genes (Silver, 2017; Falagas et al., 2019). Our results provide new insights into the critical impact of metabolic pathways, such as that of isoprenoid biosynthesis, on the antibiotic efficacy or evasion by bacteria, although more work is needed to clarify the clinical relevance. Nevertheless, improvements in our understanding of bacterial metabolism or the physiological changes occurring in the context of antibiotic treatment should help to overcome antibiotic defense mechanisms.

## Data Availability

The original contributions presented in the study are included in the article/supplementary material, further inquiries can be directed to the corresponding author.

## Footnotes

^1^ http://imageJ.net/Contributors
